# Change of Heart: the Epitranscriptome of Small Non-coding RNAs in Heart Failure

**DOI:** 10.1007/s11897-022-00561-2

**Published:** 2022-07-25

**Authors:** Tamar Woudenberg, Nyika D. Kruyt, Paul H. A. Quax, A. Yaël Nossent

**Affiliations:** 1 Department of Surgery, Leiden University Medical Center, D6-P, PO Box 9600, 2300 RC Leiden, the Netherlands; 2 Einthoven Laboratory for Experimental Vascular Medicine, Leiden University Medical Center, Leiden, the Netherlands; 3 Department of Neurology, Leiden University Medical Center, Leiden, the Netherlands

**Keywords:** Epitranscriptome, Small noncoding RNAs, Heart failure, Chemical modifications, Post-transcriptional regulation

## Abstract

**Purpose of Review:**

Small non-coding RNAs regulate gene expression and are highly implicated in heart failure. Recently, an additional level of post-transcriptional regulation has been identified, referred to as the epitranscriptome, which encompasses the body of post-transcriptional modifications that are placed on RNA molecules. In this review, we summarize the current knowledge on the small non-coding RNA epitranscriptome in heart failure.

**Recent Findings:**

With the rise of new methods to study RNA modifications, epitranscriptome research has begun to take flight. Over the past 3 years, the number of publications on the epitranscriptome in heart failure has significantly increased, and we expect many more highly relevant publications to come out over the next few years.

**Summary:**

Currently, at least six modifications on small non-coding RNAs have been investigated in heart failure-relevant studies, namely N6-adenosine, N5-cytosine and N7-guanosine methylation, 2’-*O*-ribose-methylation, adenosine-to-inosine editing, and isomiRs. Their potential role in heart failure is discussed.

## Introduction

Heart failure (HF) affects more than 64 million people worldwide [1]. HF is a heterogeneous condition, defined as the inability of the heart to circulate sufficient amounts of the blood to meet the body’s demand. Generally, HF is classified according to the ejection fraction as a measure of cardiac function during the systolic and diastolic phase: heart failure with reduced ejection fraction (HF-REF) and with preserved ejection fraction (HF-PEF). The two types differ in symptomatology, epidemiology, and pathophysiology. In HF-REF, the contractility of the left ventricle is impaired by eccentric remodeling with subsequent progressive chamber dilation, leading to a volume overload of the ventricle. This is often caused by coronary artery disease, cardiomyopathies, or heart valve disease [2, 3]. HF-PEF is characterized by concentric remodeling or ventricular hypertrophy, impairing ventricle relaxation, hampering sufficient filling of the ventricle, subsequently leading to pressure overload. Causes of HF-PEF include arterial hypertension and hypertrophic cardiomyopathy [2, 3].

As HF is a major problem in health care worldwide, research into the different physiological, cellular, and molecular aspects of HF is of utmost importance and several animal models have been developed for this purpose. The most commonly used murine model for inducing heart failure is surgical transverse aortic constriction (TAC). In this model, the aorta is partially ligated, inducing increased intracardiac pressure and therefore leading to a pressure overload [4]. Sometimes continuous infusion of angiotensin II is used to mimic chronic hypertension due to neurohumoral activation that is seen in HF patients [5]. Furthermore, congestive heart failure can be induced by injections of cardiotoxic doxorubicin, which increases calcium load in cardiomyocytes, and thereby promotes apoptosis and cardiac dysfunction [6]. To mimic ischemia-induced HF, myocardial ischemia is induced by coronary artery ligation, while volume overload is mimicked by the creation of a shunt between the arterial and venous system, or by mitral valve regurgitation [7]. As heart failure is typically associated with cardiac remodeling and cardiac hypertrophy and fibrosis play a crucial role in this, in vitro models for these processes are commonly used in heart failure research, as well.

### Small Non-coding RNAs

A frequently observed feature of HF is fetal gene expression reprogramming and, in recent years, it has been established that several classes of non-coding RNAs (ncRNAs) control this cardiac gene reprogramming in a post-transcriptional manner [8, 9]. Long non-coding RNAs (lncRNAs) and microRNAs are important examples of such ncRNAs. lncRNAs are typically longer than 200 nucleotides and possess the ability to regulate protein levels. They exert their function primarily in the cell nucleus and regulate gene expression at the transcriptional level by chromatin remodeling and transcriptional activation or interference [10]. Outside the nucleus, these lncRNAs can also affect RNA stability and decay, and messenger RNA (mRNA) translation. The role of lncRNAs in HF has been widely studied and reviewed elsewhere [11–14]. Therefore, we here focus mostly on small (< 200 nucleotides) regulatory ncRNAs.

MicroRNAs are small non-coding RNAs (~ 22 nucleotides long) that regulate protein levels by inhibiting mRNA translation and/or promoting mRNA decay. Recognition of target mRNA occurs via base pairing of the microRNA’s seed sequence, from the 2nd until the 8th nucleotide from the 5’-end of the microRNA, and a complementary sequence in the mRNA transcript, usually located in the 3’ untranslated region (UTR) [15]. Functional, mature microRNA transcripts are loaded onto Argonaute proteins to form an RNA-induced silencing complex (RISC), through which inhibition of translation or mRNA decay is initiated [16]. One microRNA can target multiple mRNAs, and microRNAs can thereby regulate complex multifactorial processes, such as cardiac remodeling [17–19]. The important role of microRNAs in heart failure has already been extensively reviewed [20–23].

Other classes of small ncRNAs that can play a role in HF include piwi-interacting RNAs (piRNAs), transfer RNA derived fragments (tRFs), and small nucleolar RNAs (snoRNAs), which all have the ability to regulate post-transcriptional gene expression. piRNAs are typically 26–31 nucleotides long and interact with the piwi-subfamily of Argonaute proteins, resulting in the formation of a RISC complex [24]. While microRNAs are involved in silencing of mRNAs, piRNAs play a role in transposon silencing [24, 25]. Cleavage of mature transfer RNAs (tRNAs) results in the formation of tRFs of various lengths ranging from 15 to 50 nucleotides. The cleavage site determines the length and sequence of the fragment and is often guided by modifications of the parent tRNA [26]. tRFs reportedly have diverse functions which include regulation of protein translation, silencing of mRNA, and regulation of transposon activity [27], and potentially play a role in cardiac hypertrophy [28]. SnoRNAs are somewhat longer transcripts, between 60 and 300 nucleotides long, and are believed to mediate chemical modifications of other RNA transcripts [29]. This facilitates ribosomal RNA (rRNA) processing, but may also affect (alternative) mRNA splicing [30].

### Epitranscriptome

Recently, an additional level of post-transcriptional regulation has been identified, referred to as the epitranscriptome. The epitranscriptome describes the body of post-transcriptional modifications that are placed on RNA molecules. Although long understudied, recent studies have demonstrated important regulatory roles for biochemical modifications of RNA [31], and currently, more than 170 different types of RNA modifications have been identified [32]. These biochemical modifications can occur on all four RNA bases, as well as on the ribose. The presence of these modifications can affect RNA structure, processing, degradation, and function. While tRNAs and rRNAs are likely the most heavily modified RNA species, it is now believed that RNA modifications occur on all RNA species. At least 24 different modifications have been identified on small ncRNAs, with the help of improved technologies such as more sensitive mass spectrometry and antibodies against modifications [33, 34]. The most common modifications identified on small ncRNAs include some form of methylation, such as N6-adenosine methylation (m6A), N5-cytosine methylation (m5C), N7-guanosine methylation (m7G), which are all methylations occurring on RNA bases, and 2’-*O*-ribose-methylation (2’Ome), which occurs on the ribose. Besides methylation, nucleotide editing in the form of adenosine deamination, including adenosine-to-inosine (A-to-I) editing and cytidine-to-uridine (C-to-U) editing, is a widespread phenomenon in microRNAs [35]. Lastly, alternative cleavage of RNA transcripts can lead to the alteration of the canonical RNA sequence, consequently affecting RNA stability or function.

In this review, we summarize the current knowledge on small ncRNA epitranscriptomic pathways in heart failure. Epitranscriptome research, especially in the cardiovascular field, is still in its infancy, and therefore, we will focus on the few RNA modifications, on which heart-relevant studies have been performed, namely m6A, m5C, m7G, 2’Ome, A-to-I editing, and isomiRs.

## N6-Methyladenosine (m6A)

Methylation of the N6 position of adenosine (m6A) is one of the most prevalent post-transcriptional modifications of RNA in eukaryotic cells [36]. RNA m6A levels are dynamically regulated by so-called m6A-writers (methyltransferases), installing m6A on RNA, and m6A-erasers (demethylases), removing m6A [37]. Changes in expression of these enzymes have been associated with HF, as well as with cardiac hypertrophy and cardiac fibrosis [38–41]. Writer enzyme METTL3 (methyltransferase like 3) is upregulated in cardiac fibrotic tissue of mice and in cardiac fibroblasts treated with TFG-β1 (transforming growth factor beta 1) [39]. Overexpression of this enzyme promoted cardiomyocyte hypertrophy, cardiac fibroblast proliferation, and collagen accumulation by affecting expression and m6A levels of collagen-related genes [38, 39]. The loss of function of writer enzyme METTL5 (methyltransferase like 5) promoted pressure overload-induced cardiomyocyte hypertrophy and adverse remodeling in mice that underwent transverse aortic constriction (TAC) by modulating histone methyltransferase activity through translational repression of SUZ12 [42]. Expression of eraser enzyme FTO (fat mass and obesity associated protein) was decreased in failing mammalian hearts and hypoxic cardiomyocytes accompanied by increased m6A in RNA [40, 41], and global knockout of FTO in mice severely affects cardiac function [43]. Rescuing FTO expression in failing mouse hearts decreased m6A levels and increased cardiac contractile function [41].

From these studies, it becomes clear that regulation of writer and eraser enzymes has the ability to control the progression of heart failure. In 2020, Gao et al. identified cardiac-hypertrophy-associated piRNA (CHAPIR), a piRNA that is abundantly expressed during cardiac hypertrophy, as a regulator of METTL3 activity. CHAPIR was able to regulate hypertrophy through direct binding of METTL3, thereby inhibiting its activity [44•]. Expression or activity of writer and eraser enzymes affects total RNA-m6A levels, and an upregulation of m6A levels was observed in human failing hearts [45] and human cardiomyopathy [46], in mice with heart failure due to pressure overload induced by TAC [40, 47••], and in rat neonatal cardiomyocytes after hypertrophic stimulation [38]. It is obvious that m6A methylation is highly implicated in heart failure. However, previously mentioned studies focus mainly on global m6A status. The implication of m6A on specific small ncRNAs in heart failure remains understudied, even though small ncRNAs show great potential as regulators of cellular processes.

For instance, changes in methylation status have been shown to affect RNA folding and structure or regulate RNA processing [48–50]*.* M6A is detected on snoRNAs, lncRNAs (including back splice variants called circular RNAs), microRNAs, and other ncRNAs [51–53]. The methylation of microRNAs can affect cellular function and fate, as m6A methylation of a microRNA can stimulate microRNA maturation, and m6A methylation in the seed sequence can affect downstream target repression [53–55]. The seed sequence consists of the 2nd nucleotide until the 8th nucleotide from the 5’-end of the microRNA and enables target recognition through complementary base pairing [15]. Our group demonstrated that m6A methylation of two different adenosine residues in seed of the vasoactive microRNA miR-494-3p (m6A1 and m6A2) influences target repression, in a manner dependent on the exact site of the m6A-mark. m6A2-miR-494-3p seemed to enhance target repression, while m6A1-miR-494-3p significantly decreased target repression of previously confirmed miR-494-3p targets, compared to unmethylated miR-494-3p. The canonical version of this microRNA is reported to be decreased in the blood circulation of HF-PEF patients [56], but implications of m6A methylation of this microRNA in a cardiac setting are still unknown.

Potential effects of m6A methylation on other small ncRNAs in a cardiac setting are lacking/limited. However, m6A methylation of lncRNAs can alter their local structure, enabling the binding of m6A-reader proteins which consequently influences abundance or function of the lncRNA [57]. Liu et al. (2013) found that MALAT1 (metastasis-associated lung adenocarcinoma transcript 1), a lncRNA that has been associated with cardiomyopathy and myocardial infarction [58], also contains a number of m6A editing sites, of which two are located in the stems of the hairpin structure of MALAT1. The methylation of these two residues likely facilitates increased interactions with m6A readers [59]. Where canonical MALAT1 regulates cardiomyocyte apoptosis [58], the implications of MALAT1-m6A in a cardiac setting have not been studied yet. However, it was recently discovered in cancer research that MALAT1-m6A appeared crucial for the migratory ability of cancer cells, as MALAT1 lacking m6A significantly suppressed the metastatic potential of cancer cells [60]. This indicates that m6A modification of lncRNAs can drastically alter cellular functions in human disease.

## N5-Methylcytosine (m5C)

The modification of cytosine into N5-methylcytosine (m5C) is well established as a DNA modification in the epigenome [61, 62]. However, this modification has also been identified on mRNAs and even ncRNAs, including tRNAs, rRNAs, and long non-coding RNAs (lncRNAs) [63, 64]. The m5C modification on RNA is placed by members of the NSUN protein family (nucleolar protein/sun RNA methyltransferase family) and DNMT2 (DNA methyltransferase-2) [65]. Some of the NSUN enzymes appear essential as Metodiev et al. (2014) showed that NSUN4 inactivation in mitochondria of mice resulted in embryonic lethality and conditional knockout of the enzyme in the heart caused cardiomyopathy [66]. In contrast, DNMT2 is not essential. However, constitutive *Dnmt2*-deficiency in mice led to cardiac hypertrophy, even though cardiac function was preserved, despite the enlargement of cardiomyocytes [67•].

The distribution of modified cytosines along the RNA is not random; CG-rich regions are enriched in m5C modifications, as are regions immediately downstream of translation initiation sites, untranslated regions, and regions near Argonaute binding sites [63, 64]. Furthermore, the m5C modification is dynamically regulated, tissue specific, and conserved in mammals [64]*.* In the heart, m5C was especially enriched in mRNAs involved in heart and muscle cell development and in mitochondrial function [68].

The effect of m5C modifications on small ncRNA in heart failure remains understudied, but in 2016, Ghanbarian et al. found a link between *Dnmt2*-deficiency induced cardiac hypertrophy and the small nuclear RNA (snRNA) Rn7sk [67•]. For transcription of protein-coding genes, regulation of the activity of RNA polymerase II is essential. This is accomplished by phosphorylation of the carboxy-terminal domain by P-TEFb (positive transcription elongation factor b). The activation of the P-TEFb complex is a critical step for hypertrophic cardiac growth [69], but Rn7sk associates with P-TEFb as a negative regulator, inhibiting its activity [70, 71]. Ghanbarian and colleagues elegantly showed that Rn7sk is significantly less methylated in Dnmt2-deficient cardiac cells and that the snRNA was significantly less associated with the P-TEFb complex compared to wild-type cells, leading to increased activation of P-TEFb and therefore causing an upregulation in hypertrophic associated genes.

The mechanism behind this increased dissociation may toile in the fact that unmethylated Rn7sk appears less stable and is therefore more readily degraded. Methylation by DNMT2 has proven to protect tRNAs from stress-induced cleavage, interfering with the formation of tRFs [26]. Blanco et al. (2014) also report an accumulation of 5’-tRNA fragments in the absence of methyltransferase NSUN2 [72]. This is merely an example of the effect of one specific modification on tRNA stability and fragmentation, while tRNAs are known to be heavily decorated with many modifications that carry the same potential. Kuscu et al. (2018) show in turn that tRNA fragments can be processed into smaller RNAs by the DICER RNase, which can then associate with Argonaute proteins and act as post-transcriptional repressor of mRNA through sequence complementarity [73]. This implies that altered tRNA cleavage due to stress or disease conditions and an altered methylation state could have phenotypic consequences.

Shen et al. (2018) looked into the role of these tRFs in cardiac hypertrophy and demonstrated, by small RNA sequencing, that tRFs were extremely enriched in isoproterenol-induced hypertrophic rat hearts. Interestingly, the cleavage patterns indicated that the induced fragments were results of precise cleavage modulations in response to myocardial hypertrophy [74••]. Overexpression of the two most prevalent fragments, 5’-Gly-CCC-30nt and 5’ Glu-TCC-34nt, increased levels of hypertrophic markers ANF (atrial natriuretic peptide), BNP (brain natriuretic peptide) and b-MHC, and cells showed significantly larger cell surface areas. Additionally, the authors demonstrated that 5’-Gly-CCC-30nt is able to directly target the 3’-UTR of Timp3 (TIMP metallopeptidase inhibitor 3), an important regulator in cardiac fibrosis and hypertrophy [75], in a microRNA-like fashion thereby proposing a mechanism for the observed effects of tRFs on cardiac hypertrophy.

## N7-Methylguanosine (m7G)

In m7G, guanosine is methylated at the 7th position of its base ring. This modification is ubiquitous in mRNA 5’caps, but also widely occurs in tRNAs and rRNAs [76, 77]. More recently, this modification was also identified in microRNAs [78•]. In 2019, Pandolifini et al. identified m7G within a subset of microRNAs, among which members of the let-7 microRNA family. M7G methylation within these microRNAs was mediated by methyltransferase METTL1 and appeared to affect their biogenesis. The authors demonstrated that the presence of m7G in G-rich regions hampers the formation of G-quadruplexes; secondary structures within RNA caused by alternative Hoogsteen base pairing of guanines in G-rich regions. The presence of these structures in microRNAs is known to inhibit microRNA biogenesis [79]. Indeed, the absence of these G-quadruplexes due to m7G caused an increase in let-7e processing.

Members of the affected let-7 microRNA family are involved in cardiac hypertrophy and heart failure, although they appear to have a dual role [80–82]. On the one hand, Satoh et al. (2011) identified that let-7i is downregulated in patients with dilated cardiomyopathy and that low let-7i levels were associated with poor clinical outcome including heart failure [80]. Furthermore, Yang et al. (2011) observed an upregulation of several let-7 microRNA family members in cardiomyocytes of mice hearts treated with angiotensin II as a model for pressure overload [81]. The authors found a link between Trx1 (Thioredoxin 1), a small redox protein that inhibits cardiac hypertrophy, and the expression levels of let-7 family members. Trx1 upregulates the expression of let-7 family members in the heart and cardiomyocytes. Knockdown of the microRNA family not only ameliorated angiotensin II-induced cardiac hypertrophy, but it also attenuated Trx1-mediated inhibition of angiotensin II-induced cardiac hypertrophy, indicating a cardioprotective effect for let-7 microRNAs in cardiac hypertrophy.

On the other hand, Tolonen et al. (2014) investigated the effect of inhibiting let-7c on the progression of postinfarction left ventricular (LV) remodeling in mice [82]. By inhibiting let-7c, expression levels of other closely related members of the let-7 family also decreased in the heart. This inhibition resulted in upregulation of pluripotency-associated genes and prevented the deterioration of cardiac function postinfarction. This indicates that inhibiting let-7 microRNAs may actually be beneficial for the prevention of postinfarction LV remodeling and the progression of heart failure. Taken together, let-7 microRNAs can play an important role in the prevention of heart failure, and m7G methylation may act as an important switch in their expression levels.

## 2’-*O*-Ribose Methylation (2’Ome)

2’Ome can occur on all four ribonucleosides of RNA and is carried out by methyltransferases like fibrillarin. During 2’Ome, a methyl group is added to the 2’ hydroxyl of the ribose moiety of a nucleoside. Most of these 2’Ome-events are guided by so-called C/D box snoRNAs [29]. Our group examined snoRNA expression in human failing hearts and found that snoRNA expression from the DLK1-DIO3 locus on human chromosome 14q32 was highest in human end-stage heart failure samples compared to naive vena saphena magna tissues and failed coronary bypasses [83•]. Even though their precise RNA targets are still unknown, these 14q32 snoRNAs predominantly bound fibrillarin, the methyltransferase involved in 2’Ome, indicating a role for snoRNA-guided 2’Ome in the progression of heart failure. This was supported by James et al. (2021), who identified altered snoRNA cargo in extracellular vesicles excreted by human-induced pluripotent stem cells differentiated into cardiomyocytes, that were stimulated with increased contractile workload to simulate hypertrophic cardiomyopathy [84•]. These same cardiomyocytes exhibited differential splicing of RNA within known hypertrophic cardiomyopathy related pathways and further increasing contractile workload aggravated this.

Our group confirmed 2’Ome in six integrin pathway mRNAs by a human 14q32 snoRNA, SNORD-113–6. The affected mRNAs showed decreased methylation and increased degradation when SNORD-113–6 was inhibited in human primary fibroblasts [85••]. As reviewed by Civitarese and colleagues (2017), integrins and disrupted integrin signaling between cardiac fibroblasts and cardiomyocytes contribute to cardiac fibrosis and cardiac hypertrophy [86], suggesting altered integrin signaling by 2’Ome may influence the clinical progression of heart failure.

2’Ome is omnipresent in rRNA, tRNA, and mRNA but has also been observed in small ncRNAs, including microRNAs and even snoRNAs themselves [87, 88], but its effect has not yet been studied in a cardiac setting. 2’Ome was suggested to protect adenosine residues from A-to-I editing in some in vitro studies [89, 90]. However, both 2’Ome and A-to-I were increased simultaneously in a vasoactive microRNA, also transcribed from the DLK1-DIO3 locus, under ischemia in a hindlimb ischemia mouse model, challenging this suggestion [88].

## A-to-I Editing

Where methylation mostly affects RNA stability and binding efficacy, RNA editing directly changes the nucleotide sequence. A-to-I editing is catalyzed by adenosine deaminase acting on RNA 1 or 2 (ADAR1 or ADAR2). Although the expression of both ADAR enzymes and the number of A-to-I edited sites in the heart appear to be rather low compared to other tissues [91, 92], they have proven to be crucial for cardiomyocyte survival. ADAR1 was found to be essential for normal embryonic cardiac growth and development [93] and cardiomyocyte specific deletion of ADAR1 caused severe cardiac dysfunction and increased lethality in mice [94]. ADAR2 was found to be increased in exercised hearts, protecting them against cardiac injury after myocardial infarction [95]. Furthermore, a strong downregulation of ADAR2, but upregulation of ADAR1, was observed in blood samples from congenital heart disease (CHD) patients [96].

In A-to-I editing, adenosine is deaminated, which results in the formation of an inosine. Inosine is interpreted as a guanosine by the cellular machinery, as inosine preferentially binds cytidine [97]. For mRNAs, this can result in a destabilization of its secondary structure, or an alteration in protein amino acid sequence due to an alteration in de coding sequence, that can impact cardiovascular health [98, 99]. For microRNAs, A-to-I editing can drastically impact microRNA biogenesis, target mRNA selection, and silencing efficacy [35, 100, 101].

MicroRNAs are edited in their primary transcripts, as ADARs preferentially target double-stranded RNA structures in the nucleus [102]. The editing of a primary microRNA can either enhance or hamper cleavage efficiency of ribonucleases DROSHA and DICER, and can therefore interfere with microRNA maturation [100, 101]. When editing occurs in the seed sequence of the microRNA, however, this can lead to a complete change of the mature microRNA’s target selection, resulting in the regulation of a different targetome [88, 103].

MicroRNA editing profiles are tissue specific [104, 105] and can be altered during pathophysiological processes [88, 106]. Unfortunately, research on the effects of A-to-I editing events of microRNAs in a cardiac context is still limited. However, our group did identify several microRNAs that had a higher or lower percentage of mature microRNA editing in the heart compared to other tissues, when looking at A-G mismatches in publicly available RNA sequencing data as an indicator for A-to-I editing [107•]. For instance, in humans, miR-411-5p showed a low incidence of editing of 7.3% in the heart versus 62.5% in the brain and 57.1% in the lungs. MiR-376a + b-3p, on the other hand, showed increased editing in the heart compared to the brain and lungs (94.8% versus 76.5% and 81.7%, respectively). In both microRNAs, editing occurred in the seed sequence, and the effects of these events were investigated in a vascular setting. Both edited microRNAs were able to repress a unique set of targets compared to their canonical counterpart. The targetomes of edited miR-376a + b-3p and “wild-type” miR-411-5p encompassed mostly targets involved in Wnt- and Cadherin-signaling, while wildtype miR-376a + b-3p and edited miR-411-5p did not. Both Wnt- and Cadherin-signaling pathways are highly implicated in the functionality of intercalated discs (ICDs), structures that connect neighboring cardiomyocytes in the heart [108]. Disrupted ICD-related signal transduction leads to myocardium remodeling and ultimately to heart failure, implying that changes in editing events of miR-367a + b-3p and miR-411-5p have the potential to substantially affect heart failure progression.

## IsomiRs

All RNAs, including ncRNAs, undergo various processing steps after their transcription. For mature microRNAs, this means that they are generated from a primary transcript, which undergoes two consecutive cleavage steps [109]. The loose ends of the hairpin-structured primary microRNA are cleaved off by the microprocessor complex [110], and after transport of the newly generated precursor microRNA from the nucleus to the cytoplasm, the terminal loop (the hairpin) is cleaved of by RNase II endonuclease Dicer and related cofactors [111, 112]. From the resulting microRNA duplex, either side can become a functional mature microRNA [113]. Small variations in cleavage site selection by either the microprocessor complex or Dicer can lead to altered 3’- or 5’-ends of the mature microRNA, generating isoforms that differ in length or sequence compared to their canonical version, which are called 3’- or 5’-isomiRs, respectively [114, 115]. 3’-isomiRs can also arise due to cleavage of single to several nucleotides by exonucleases or by addition of nucleotides by nucleotidyl-transferases [116].

IsomiRs are proven to be functional microRNAs that actively associate with the RISC complex and inhibit mRNA translation of their targets [115, 117, 118]. Moreover, their expression patterns are cell and tissue specific [119]. Using deep sequencing, distinctive isomiR expression profiles were found for murine cardiomyocytes and the rat heart left ventricle [120••, 121]. Approximately 50% of the microRNA annotations found in these cells and tissue accounted for isomiRs, with several isomiRs showing a higher expression than their canonical counterparts. Most variations could be found at the 3’-end of microRNAs (48.7–60.1%), but also isomiRs with changes at the 5’-end were identified (2.9–7.8%). While 3’-isomiRs have been associated with altered microRNA stability and turnover, 5’-isomiRs are associated with shifts in targetome [117, 122].

Humphreys et al. (2012) identified that the most frequently annotated microRNA sequence that was attributed to miR-133a in murine cardiomyocytes was not the canonical sequence, but a 5’-isomiR [120••]. That same 5’-isomiR appeared to be the most abundant form of miR-133a in the rat heart left ventricle in a deep sequencing study performed by McGahon et al. (2013) [121]. As a microRNA’s 5’-end determines its seed sequence, and therefore, its target pool, a shift in seed sequence due to an addition or deletion of a nucleotide, will affect microRNA target selection [15]. This was demonstrated by Humphreys et al. using a reporter gene assay; the canonical miR-133a was able to induce a significantly greater repression of the *Ctgf* (connective tissue growth factor) target reporter than the 5’-isomiR, while the 5’-isomiR repressed the *Pgam1* (phosphoglycerate mutase 1) target reporter better than canonical miR-133a.

MiR-133a has been identified as a major cardiac regulator and is highly implicated in heart failure [123]. A decrease in expression of miR-133a is observed in several rodent models of heart failure [123–127], but also in the hearts of patients with hypertrophic cardiomyopathy or chronic heart failure [123, 125, 127]. Overexpression of this microRNA protects against cardiac hypertrophy [123, 125] and fibrosis of the heart [124, 128] in rodents. Carè et al. (2007) and Duisters et al. (2009) identified *RhoA* and *Cdc42*, proteins implicated in the hypertrophic signaling cascade, and *Ctfg*, implicated in cardiac fibrosis, as direct targets of miR-133a, proposing a mechanism through which miR-133a exerts its protective effects [123, 127]. Keeping in mind the results by Humphreys et al. regarding the reduced repression of *Ctfg* by the more abundantly expressed 5’-isomiR-133a as mentioned above, the isomiR may prove clinically relevant in heart failure.

However, so far, no one has looked into the effects of this highly abundant 5’isomiR of miR-133a in heart failure. The difference in target selection by isoforms of a microRNA can lead to differences in biological functions. Our group previously demonstrated that the 5’-isomiR of the known vasoactive miR-411-5p is highly expressed in the heart and vasculature. The 5-isoforms of miR-411-5p have highly distinctive targetomes and were shown to be are differentially regulated under ischemia [129•]. Under normoxic conditions, the isomiR/canonical microRNA ratio exerts anti-angiogenic effects, which shifts towards pro-angiogenic activity under hypoxic conditions. This indicates that the body can regulate or react to pathophysiological processes by controlling isomiR levels. MiR-411-5p seems to be involved in heart failure as well as a decrease of miR-411-5p was observed in neonatal rabbits with left ventricle pressure overload induced by TAC [130]. Additionally, miR-411-5p is suggested to induce proliferation in adult murine cardiomyocytes via modulation of the Hippo signaling pathway [131]. However, the role of its 5’-isomiR in heart failure progression is still unknown.

## Conclusion

In conclusion, the available literature shows that post-transcriptional modifications of small non-coding RNAs are likely to play important roles in the regulation of gene expression of many factors involved in the occurrence, progression, and regulation of heart failure. While epitranscriptome research in a cardiac context is still in its infancy, we observed an increase in relevant publications over the past 3 years. The rise of new methods to study RNA modifications is expected to facilitate an exponential increase in more in-depth research concerning the role of the epitranscriptome in heart failure. Given the fact that even with the limited amount of research available now, the implications of the epitranscriptome for heart failure are significant; we can only anticipate exciting and highly important research developments over the next few years.
